# Complete Genome Sequence of the Live Attenuated Vaccine Strain Brucella melitensis Rev.1

**DOI:** 10.1128/genomeA.00175-18

**Published:** 2018-03-22

**Authors:** Mali Salmon-Divon, Menachem Banai, Svetlana Bardenstein, Shlomo E. Blum, David Kornspan

**Affiliations:** a Genomic Bioinformatics Laboratory, Department of Molecular Biology, Ariel University, Ariel, Israel; b Department of Bacteriology, Kimron Veterinary Institute, Bet Dagan, Israel

## Abstract

Live attenuated vaccines are essential elements in control programs for the prevention of brucellosis. Here, we report the whole-genome sequence of the original Elberg Brucella melitensis Rev.1 vaccine strain, passage 101 (1970). Commercial lines of the original strain have been successfully used in small ruminants worldwide.

## GENOME ANNOUNCEMENT

Brucellosis is the most common bacterial zoonotic disease worldwide, infecting over half a million people annually (1). Brucella species are intracellular Gram-negative bacteria which were first isolated in 1887 by Sir David Bruce from the spleens of soldiers with fatal cases of brucellosis (1). Among the six classically known Brucella species, B. melitensis is considered the most pathogenic species in humans (2). Brucella species cause abortions in the third trimester in their natural hosts (3), and live attenuated vaccines are aimed at preventing these events in order to reduce the environmental bacterial load. B. melitensis vaccine strain Rev.1 was developed by Elberg and Herzberg in the mid-1950s (4) and was shown to successfully protect and reduce abortions among small ruminants (5). Unlike B. melitensis type strains, Rev.1 is resistant to streptomycin and susceptible to penicillin G (4, 5). In 1970, passage 101 was made available as a freeze-dried seed stock culture. The strain originating from this passage resembled the original parental seed material, making it compatible for prophylactic vaccination of small ruminants (5). One ampule of the lyophilized stock was sent to Menachem Banai (5), at the Israeli National, OIE and FAO Reference Laboratory for Brucellosis, Kimron Veterinary Institute, Israel, following his personal correspondence with Sanford Elberg (4). An ampule of the same Rev.1 seed material was sent simultaneously to Jean-Michel Verger, INRA, who further reestablished its innocuousness and deposited the strain in European Pharmacopoeia (6). Here, we report a whole-genome sequence of this original strain.

Genomic DNA was purified using the DNeasy blood and tissue kit (Qiagen) and was sent for sequencing in both PacBio and Illumina platforms (Institute for Genome Sciences, Baltimore, MD, and Crown Institute for Genomics, G-INCPM, Weizmann Institute of Science, Israel, respectively). The average read length of the PacBio raw data was ∼7 kb, with a maximum read length of about 42,000 bases (coverage, ∼496×). Illumina paired-end sequencing generated 1.2 million 2 × 250-bp reads (coverage, ∼182×). Hybrid assembly of the genome using both short (Illumina) and long (PacBio) reads was done using the Unicycler pipeline (7).

The genome assembly produced two large scaffolds of 2,121,368 and 1,177,802 bp in length, representing the two B. melitensis chromosomes. The sizes of the chromosomes were highly similar to those of the 16M reference strain (2,117,144 and 1,177,787 bp), generating an estimated total genome size of 3,299,170 bp. Genome annotation was carried out using the Rapid Annotations using Subsystems Technology (RAST) server (8). The total genome had a G+C content of 57.2% and contained 3,327 open reading frames (ORFs), 54 tRNAs, and 9 rRNA genes. Out of the 3,327 coding sequences, 1,741 were categorized into RAST subsystems. Within the 1,741 annotated coding sequences (CDSs), the majority were classified into subsystems of amino acids and derivatives (429 CDSs), carbohydrates (367 CDSs), cofactors, vitamins, prosthetic groups, pigments (265 CDSs), protein metabolism (246 CDSs), and membrane transport (161 CDSs). A further detailed analysis will be included in our future publication.

### Accession number(s).

This whole-genome shotgun project has been deposited in DDBJ/EMBL/GenBank under the accession numbers CP024715 and CP024716.
